# Reliable Fiber Sensor System with Star-Ring-Bus Architecture

**DOI:** 10.3390/s100504194

**Published:** 2010-04-27

**Authors:** Peng-Chun Peng, Jun-Bo Wang, Kuan-Yan Huang

**Affiliations:** 1 Department of Electro-Optical Engineering, National Taipei University of Technology, Taipei, Taiwan; E-Mail: aupt1215@hotmail.com; 2 Department of Electrical Engineering, National Chi Nan University, Nantou County, Taiwan; E-Mail: hky_tw@hotmail.com

**Keywords:** sensor system, star-ring-bus architecture, fiber Bragg grating

## Abstract

This work presents a novel star-ring-bus sensor system and demonstrates its effectiveness. The main trunk of the proposed sensor system is a star topology and the sensing branches comprise a series of bus subnets. Any weakness in the reliability of the sensor system is overcome by adding remote nodes and switches to the ring and bus subnets. To construct the proposed star-ring-bus sensor system, a fiber ring laser scheme is used to improve the signal-to-noise ratio of the sensor system. The proposed system increases the reliability and capacity of fiber sensor systems.

## 1. Introduction

Fiber grating sensors are important sensing elements [1,2]. They are effective for strain [3], temperature [4,5], refractive index [6], and pressure [7] monitoring. The multiplexing capability is a unique feature of a fiber Bragg grating (FBG) sensor system. Techniques for multiplexing FBGs are wavelength division multiplexing [8], time division multiplexing [9], code division multiplexing [10], intensity and wavelength division multiplexing [11], and frequency modulated continuous wave multiplexing [12]. Based on these multiplexing schemes or their combinations, this work constructs a large FBG sensor system. As a result, how to enhance the reliability of FBG sensor systems becomes a significant problem, as general system architectures, such as the in-line architecture [3,13,14], bus architecture [15], star architecture [16], or tree architecture [17] cannot protect a sensor system. To ensure survivability of a fiber sensor system and protect against environmental accidents, protection schemes become essential in practical fiber sensor applications. Recently, a double-ring optical system to FBG sensors has been proposed [18]. However, when the system has more than two breakpoints, this architecture also cannot protect the FBG sensor system.

In this paper, we present a novel star-ring-bus sensor system with a reconfigurable function that increases system reliability and capacity. This reconfigurable function can be applied to remote nodes and optical switches to reconfigure the star-ring-bus system when any link fails. This simple function for a sensor system can identify a sudden breakpoint in a fiber link. Nevertheless, the drawback of the proposed reconfigurable function for the sensor system is that all switches and remote nodes in a system generate additional losses and further reduce the signal-to-noise ratio. Recently, fiber laser schemes to provide a high output power and improve the signal to noise ratio for FBG sensor systems were reported [19,20]. Moreover, the power variation of an erbium-doped fiber ring laser is below 0.55 dB from 1,528 nm to 1,572 nm [21]. Therefore, the proposed sensor system uses a fiber ring laser to improve the signal-to-noise ratio. This sensor system is implemented by tuning a tunable bandpass filter located within the cavity of fiber ring laser for analyzing Bragg wavelengths of all sensing FBGs. The Bragg wavelength shifts induced by strain drift on FBGs can be measured by identifying the lasing wavelength shifts of the fiber ring laser. Because the interrogation method relies on the tunable bandpass filter, the sensor system is suitable for static or low-frequency dynamic strain measurement. This limitation of response time can be improved by utilizing a faster tunable bandpass filter. A Fabry-Perot filter using a 16 bit digital-to-analog converter generates a minimum resolvable wavelength shift of about 0.8 pm [3]. Moreover, the filter can be scanned at rates >300 Hz. The benefits of the fiber ring laser in conjunction with the star-ring-bus architecture can facilitate the highly reliable sensor system. The sensor system is good for health monitoring of structures such as bridges, tunnels, dams and buildings because many sensors can be embedded in these structures.

## 2. Architecture

**Figure 1.**
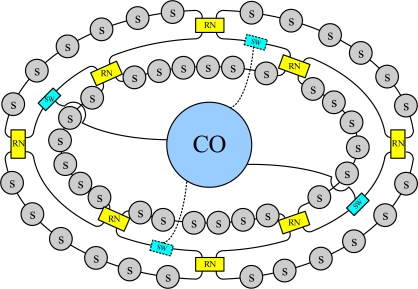
Schematic diagram of the proposed fiber sensor system. [central office (CO), remote node (RN), optical switch (SW), sensor (S)].

Figure 1 shows the proposed star-ring-bus architecture for the FBG sensor system. The star-ring-bus architecture consists of the FBG sensor network and a central office providing the light source and differentiating the sensing signals from the sensor network. The sensor network has a star subnet on its upper level as the network infrastructure, a ring subnet on the middle level, and bus subnets on the lower level, which serve various FBG sensors. The light sources are distributed to switches *via* the upper-level star network, and are then delivered to each FBG sensor through the mid-level ring subnet and bus subnets on the lower level. The remote node transfers signals between the mid-level ring subnet and lower-level bus subnets, and performs a self-healing function when a link failure occurs in the lower level of the network.

**Figure 2.**
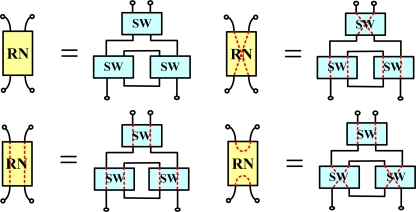
Schematic diagram of a remote node.

Each remote node has three 2 × 2 optical switches (Figure 2). Bus subnets are attached to ring subnets *via* remote nodes. The remote node in each bus subnet can be controlled by the time-division multiplexing signal to enhance sensor network capacity.

**Figure 3.**
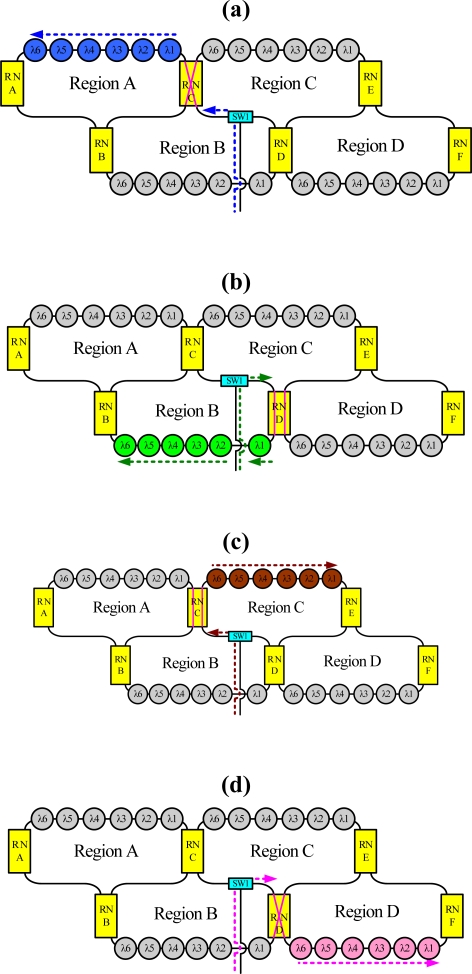
Schematic of the situations (indicated by the dashed line) (a) when sensing Region A is selected by the time-division multiplexing signal, (b) when sensing Region B is selected by the time-division multiplexing signal, (c) when sensing Region C is selected by the time-division multiplexing signal, and (d) when sensing Region D is selected by the time-division multiplexing signal (λi is the fiber Bragg grating sensor).

**Figure 4.**
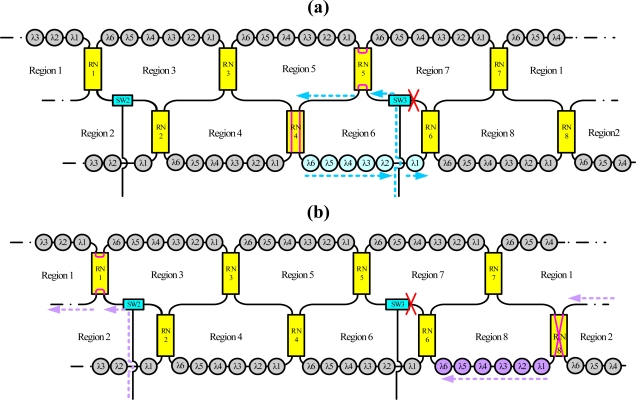
Schematic diagram breakpoints in the ring subnet.

**Figure 5.**
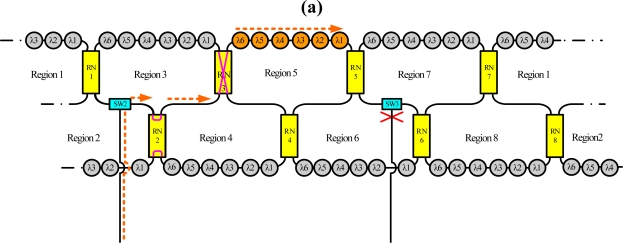

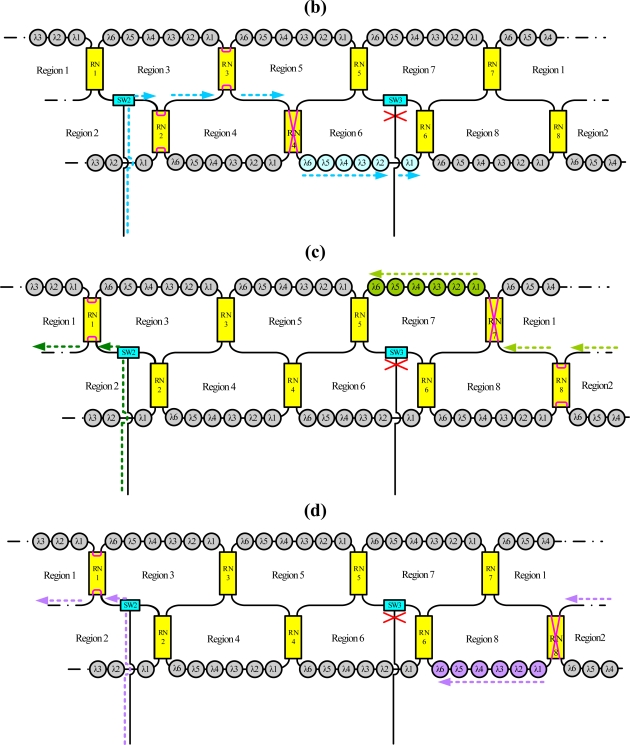
Schematic diagram of breakpoints in the star subnet.

For instance, the dashed line in Figures 3(a–d) indicates schematically the situation when sensing regions A, B, C and D are selected, respectively, using the time-division multiplexing signal. Because this bus topology is combined with the time-division multiplexing technology, the proposed system supports a large number of sensors. Moreover, for such a large sensor network, this work applies the reconfigurable function for the sensor network by controlling remote nodes and optical switches. Such an architecture offers a survival function under link failure by reconfiguring the sensor network. Figure 4 shows schematically a situation in which a fiber link in the ring subnets fails. In this case, the sensing signals from sensing Region 8 are lost. However, remote nodes 1 and 8 (RN1 and RN8) can be modified to retransmit sensing signals in sensing Region 8. If the corresponding link in the upper-level star subnet is broken, the control circuit reconfigures the remote node function (Figure 5) such that the network can be modified to retransmit the sensing signals.

**Figure 6.**
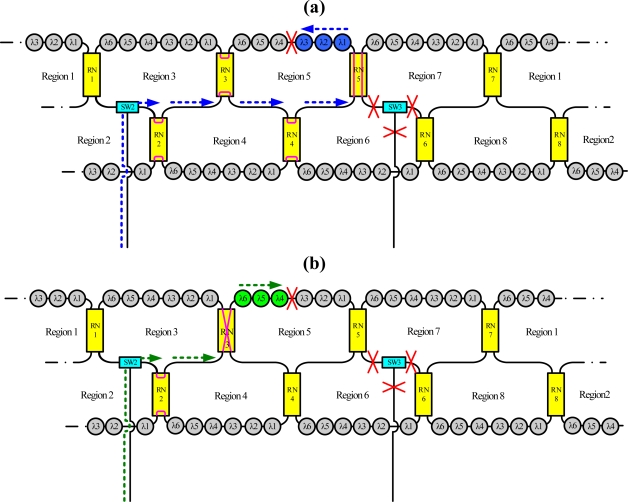
Schematic diagram of breakpoints in the bus and ring subnets.

**Figure 7.**
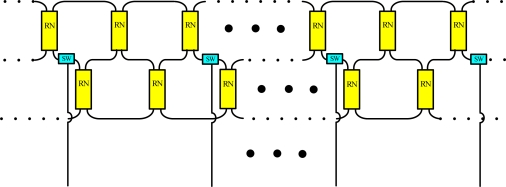
Schematic diagram of proposed sensor network.

Figure 6 shows schematically a situation in which a fiber link fails in the bus subnet and the ring subnet. In this case, the sensing signals from sensing Region 5 are lost. Nevertheless, remote nodes 3 and 5 (RN3 and RN5) can be modified to retransmit sensing signals in sensing Region 5, as shown in Figure 6. Moreover the reconfigurable function can be improved by increasing the number of remote nodes and optical switches (Figure 7).

## 3. Experimental Results

**Figure 8.**
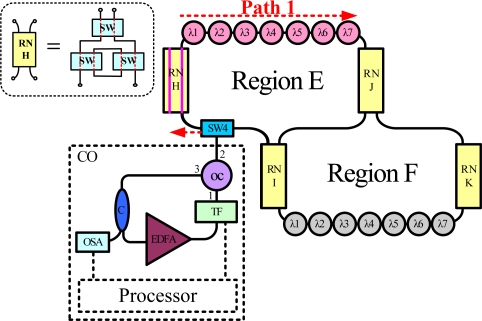
Experimental setup for proposed fiber sensor system. (tunable bandpass filter (TF), erbium-doped fiber amplifier (EDFA), optical circulator (OC), optical coupler (C), optical spectrum analyzer (OSA)).

Figure 8 shows the experimental setup for the proposed FBG sensor system. This work examined two subnets in the star-ring-bus architecture. The central office (CO) in this system has a tunable bandpass filter (TF), erbium-doped fiber amplifier (EDFA), optical circulator (OC) and optical coupler (C). The EDFA was manufactured by Infomax Optical Technology Corporation and had a saturated output power of 17 dBm. The light source in this system is a fiber laser comprising a fiber loop and the sensing FBGs (λ1, λ2,… and λ7), which simultaneously act as cavity mirror. The Bragg wavelengths of the FBGs from FBG λ1 to FBG λ7 were sequentially 1,535.95, 1,538.82, 1,542.18, 1,544.84, 1,547.92, 1,551, and 1,553.87 nm. The average reflectivity and bandwidth of the FBGs were 92% and 0.16 nm, respectively. In the central office, the lasing wavelength of the fiber laser was determined by these sensing FBGs in conjunction with the tunable bandpass filter (TF). The 3 dB bandwidth and insertion loss of this TF were 0.35 nm and 5 dB, respectively. In this fiber laser approach, the coupling ratio of the optical coupler (C) is 90:10. The lasing light from the optical coupler arrives in an optical spectrum analyzer (OSA). With sufficient gain, the system lases once the transmitted wavelength of the filter equals the wavelength reflected from the sensing FBGs. Thus, the lasing wavelength of the system can be utilized to accurately measure strain perturbation on the FBGs.

**Figure 9.**
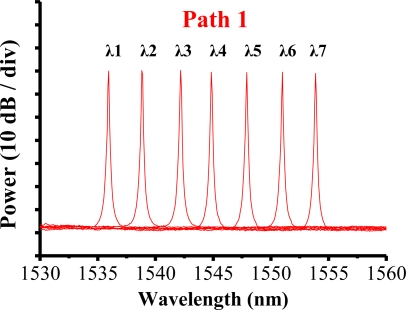
Output spectra of fiber laser at various lasing wavelengths.

**Figure 10.**
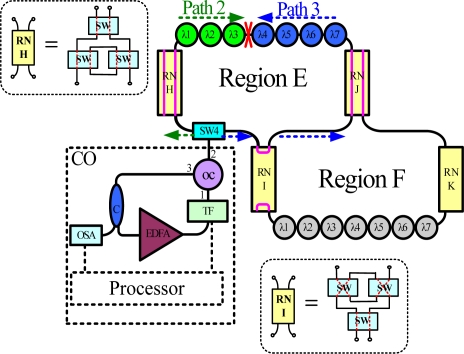
Link failure.

**Figure 11.**
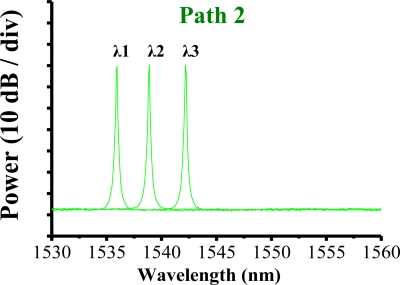
When a link fails, the FBGs λi (i = 4, 5, 6, and 7) lose their sensing signal.

**Figure 12.**
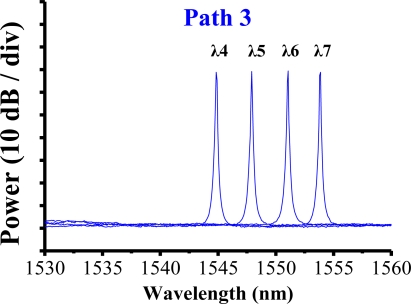
The remote node can be modified to reconfigure the fiber link for FBGs λi (i = 4, 5, 6, and 7) that lost their sensing signal.

The filter was tuned to select the transmitted wavelength over a working range of 1,535–1,565 nm. Hence, the tunable transmitted wavelength of the filter tracked the seven wavelengths from the sensing FBGs λi (i = 1, 2, …, 7). Figure 9 shows the output spectra of the fiber laser at different lasing wavelengths. The signal-to-noise ratio of the sensor system is over 65 dB. When the link failed at Region E (Figure 10), the FBGs λi (i = 4, 5, 6, and 7) lost its sensing information (Figure 11). However, when link failure occurred, remote node I (RNI) was modified to reconfigure the fiber link for FBG λi (i = 4, 5, 6, and 7). The lost sensing information can be retransmitted during link reconstruction (Figure 12). Consequently, the proposed self-healing function can reconstruct the sensor system and enhance its capacity.

In this paper, we concentrate our study on how to achieve the star-ring-bus architecture for a reliable FBG sensor system. The optical switches have an insertion loss of about 0.2∼0.6 dB and a return loss of over 60 dB. Hence, the sensing signals in Path 1, Path 2, and Path 3 have the approximate signal-to-noise ratio and power. In addition, we could use an athermal FBG strain sensor (from Fibera Inc.) to eliminate the temperature influence on the sensors. The FBG strain sensor is stable from −20 °C to 70 °C [22]. Furthermore, the frequency shift/μstrain is 0.18 GHz (1.45 pm)/μstrain. However, the crosstalk induced by the adjacent FBGs limits the number of FBG sensors. To avoid such a problem, a feedback-controlled circuitry [8] could be used in the system. For example, we choose 2.66 nm as the minimum wavelength spacing between the FBGs in our experiment. This wavelength spacing indicates that the maximum strain imposed on each FBG has to be smaller than about 1,834 μstrain [22] and this quantity should be accurately controlled by the feedback circuitry. The optimal sensor system can be designed by considering the maximum measured strain and the scale of the star-ring-bus architecture.

## 4. Conclusions

This work has presented a novel star-ring-bus sensor system. The reconfigurable function is constructed using optical switches and remote nodes. The remote nodes and optical switches in the star-bus-ring architecture are utilized to enhance sensing capacity, identify the breakpoint, and reconfigure the sensor network. This work demonstrated two subnets based on the proposed configuration and examined their network survivability. Due to the significant amount of lasing power from the fiber ring laser, the signal-to-noise ratio of the sensor system can exceed 65 dB. Results of this study demonstrate that the proposed system is a highly reliable fiber sensor system for large multipoint smart structures.
